# Supplementary material for: DMRforPairs: identifying Differentially Methylated Regions between unique samples using array based methylation profiles
Source: BMC Bioinformatics. 2014 May 15;15:141. doi: 10.1186/1471-2105-15-141 (PMC4046028; doi:10.1186/1471-2105-15-141)
Supplement: Additional file 2 — DMRforPairs output for the comparison of A431-MCF7 and NA17018-NA17105. Please start from the HTML files in each folder. Available via the BMC Bioinformatics website. [file 1471-2105-15-141-S2.zip › 1394847754114233_MOESM2_ESM/NA17105M_NA17018F/figures/35754.pdf]

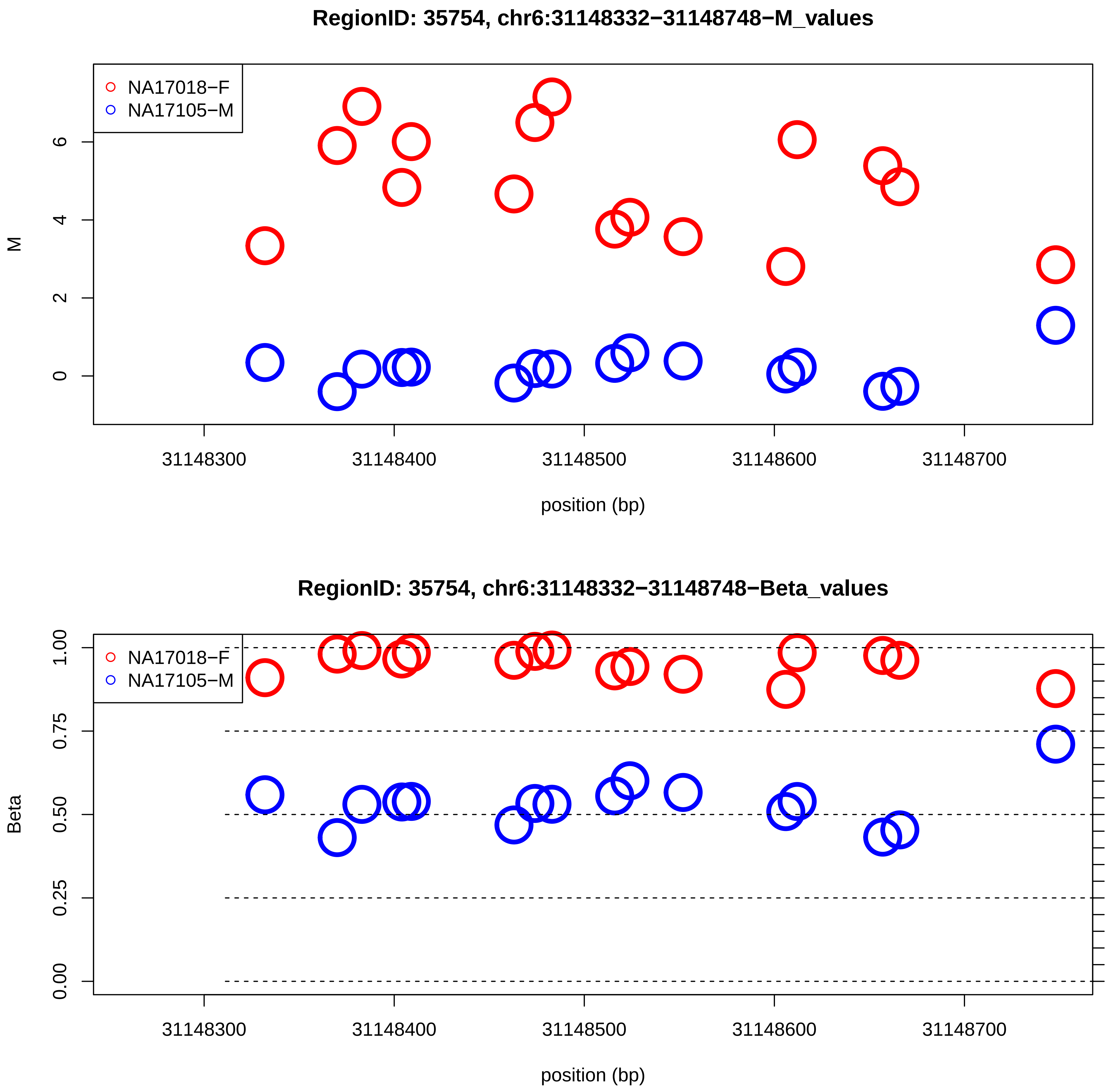
RegionID: 35754, chr6:31148332−31148748−M_values
NA17018−F
NA17105−M
M
0
2
4
6
31148300
31148400
31148500
31148600
31148700
position (bp)
RegionID: 35754, chr6:31148332−31148748−Beta_values
NA17018−F
NA17105−M
Beta
0.00
0.25
0.50
0.75
1.00
31148300
31148400
31148500
31148600
31148700
position (bp)

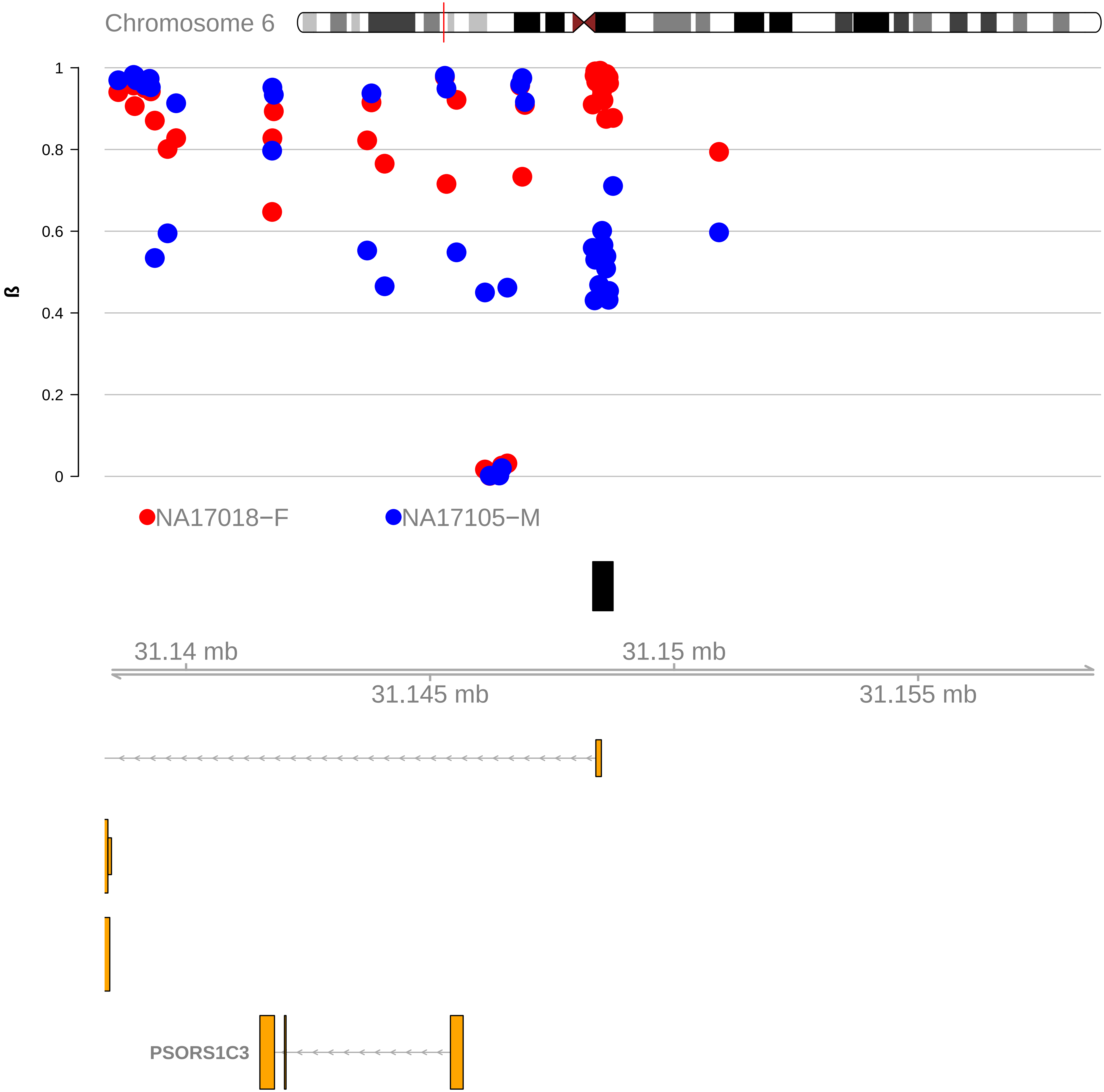
Chromosome 6
ß
1
0.8
0.6
0.4
0.2
0
NA17018−F
NA17105−M
31.14 mb
31.15 mb
31.145 mb
31.155 mb
PSORS1C3
